# *In vivo* activation of methyl-coenzyme M reductase by carbon monoxide

**DOI:** 10.3389/fmicb.2013.00069

**Published:** 2013-04-01

**Authors:** Yuzhen Zhou, Alexandria E. Dorchak, Stephen W. Ragsdale

**Affiliations:** Department of Biological Chemistry, University of Michigan Medical School, University of MichiganAnn Arbor, MI, USA

**Keywords:** carbon monoxide, enzyme catalysis, metalloenzymes, nickel, electron transfer, EPR, methanogenesis, enzyme activation

## Abstract

Methyl-coenzyme M reductase (MCR) from methanogenic archaea catalyzes the rate-limiting and final step in methane biosynthesis. Using coenzyme B as the two-electron donor, MCR reduces methyl-coenzyme M (CH_3_-SCoM) to methane and the mixed disulfide, CoBS-SCoM. MCR contains an essential redox-active nickel tetrahydrocorphinoid cofactor, Coenzyme F_430_, at its active site. The active form of the enzyme (MCR_red1_) contains Ni(I)-F_430_. Rapid and efficient conversion of MCR to MCR_red1_ is important for elucidating the enzymatic mechanism, yet this reduction is difficult because the Ni(I) state is subject to oxidative inactivation. Furthermore, no *in vitro* methods have yet been described to convert Ni(II) forms into MCR_red1_. Since 1991, it has been known that MCR_red1_ from *Methanothermobacter marburgensis* can be generated *in vivo* when cells are purged with 100% H_2_. Here we show that purging cells or cell extracts with CO can also activate MCR. The rate of *in vivo* activation by CO is about 15 times faster than by H_2_ (130 and 8 min^-1^, respectively) and CO leads to twofold higher MCR_red1_ than H_2_. Unlike H_2_-dependent activation, which exhibits a 10-h lag time, there is no lag for CO-dependent activation. Based on cyanide inhibition experiments, carbon monoxide dehydrogenase is required for the CO-dependent activation. Formate, which also is a strong reductant, cannot activate MCR in *M. marburgensis in vivo*.

## INTRODUCTION

Methanogens are responsible for all biological methane production on earth, generating 10^9^ tons of methane annually. Being strict anaerobes, they are widely distributed in anoxic environments where electron acceptors such as ${\text{NO}}_{3}^{-}$ , Fe^3+^, and ${\text{SO}}_{4}^{2-}$ are limiting, such as aquatic sediments, the digestive tract of animals, rice fields, sewage digesters, landfills, heart wood of living trees, and decomposing algal mats (Garcia et al., 2000). Methanogens play critical roles in the carbon cycle by converting products from anaerobic fermentation (such as hydrogen, carbon dioxide, methanol, formate, and acetate) into methane (Thauer, 1998; Ferry, 2010). Only by such means can methanogens obtain energy to grow (Thauer, 1998; Ferry, 2010). Methane, the principal component of natural gas, is an important source of clean renewable energy, with the highest heat production per mass unit (55.7 kJ g^-1^) among all hydrocarbons (Richard and Ball, 2008). However, methane also is a potent greenhouse gas (Hanson and Hanson, 1996) and, predominantly due to changing agricultural practices (e.g., increased development of rice production and livestock cultivation) over the past two centuries, the atmospheric concentration of methane has more than doubled, reaching a level (1770 ppb in 2005) that far exceeds the natural range (320–790 ppb) of the last 650,000 years (Wuebbles and Hayhoe, 2000). Therefore, because of the effect of greenhouse gases on climate change, understanding the basis of methane production is an important research goal, while controlling methane emissions is an important aim for governmental policy makers.

Methyl-coenzyme M reductase (MCR, EC 2.8.4.1), a nickel-containing enzyme, catalyzes the rate-limiting and final step of methane production and the first step in anaerobic methane oxidation (Dey et al., 2010b; Scheller et al., 2010). For methane formation, MCR converts methyl-coenzyme M (CH_3_-SCoM) and coenzyme B (CoBSH) to methane and the heterodisulfide of coenzyme M and coenzyme B (CoBS-SCoM) as shown in Eq. 1 (DiMarco et al., 1990; Thauer, 1998).

$$\begin{array}{cc} {\text{CH}}_{3}\text{-SCoM}+\text{CoBSH}\to {\text{CH}}_{4}+\text{CoBS-SCoM}\Delta {\text{G}}^{0^{\prime }}=-30\text{KJ }{\text{mol}}^{-1} & \left(1\right) \end{array}$$

A number of high-resolution crystal structures of MCR are available (Ermler et al., 1997; Grabarse et al., 2000, 2001; Cedervall et al., 2010, 2011). These structures reveal MCR to be a dimer of heterotrimers (α_2_β_2_γ_2_) with a molecular mass of around 270 kDa (Ermler et al., 1997). The three subunits (αβγ) are tightly associated, especially between subunits α and α′ and between β and β′, forming two 50 Å hydrophobic channels (one in each heterotrimer) ending in a pocket that accommodates a deeply embedded redox-sensitive nickel tetrapyrrole cofactor called coenzyme F_430_, which plays an essential role in catalysis (Goubeaud et al., 1997; Becker and Ragsdale, 1998). The substrates and products thread through the channel toward F_430_. In each heterotrimer, there is one F_430_-containing active site, located 50 Å from the F_430_ in the other heterotrimer. Due to its extreme O_2_ sensitivity, MCR in all of these structures but one contains F_430_ in the inactive Ni(II) state (Ermler et al., 1997; Grabarse et al., 2000, 2001; Cedervall et al., 2010), the other being methyl-Ni(III) (Cedervall et al., 2011). More than 16 distinct oxidation and coordination states of MCR have been spectroscopically characterized (Cedervall et al., 2010). To initiate catalysis, the enzyme must be in the MCR_red1_ state, which contains the redox-active nickel as Ni(I) (Albracht et al., 1988; Goubeaud et al., 1997; Becker and Ragsdale, 1998). The binding site of HSCoM (and presumably CH_3_-SCoM) is more deeply buried within the enzyme, and so it must enter prior to CoBSH for productive chemistry to occur. Therefore, it was suggested that a conformational change upon CH_3_-SCoM binding might lower the *K*_d_ for CoBSH and promote an ordered substrate binding mechanism (Grabarse et al., 2001). Furthermore, electron paramagnetic resonance (EPR) and electron nuclear double resonance (ENDOR) studies indicate that CoBSH binding induces a conformational change that brings the methyl group of methyl-SCoM into closer proximity to the nickel, which could promote C–S bond cleavage (Ebner et al., 2010).

Two main catalytic mechanisms for MCR-catalyzed reaction have been under debate for a long time. These two mechanisms in principle differ in how they describe the initial cleavage of sulfur–carbon bond of the substrate CH_3_-SCoM (Ermler, 2005). Mechanism I involves a methyl-Ni(III) intermediate (Ermler et al., 1997), while mechanism II postulates formation of a methyl radical and a CoMS–Ni(II) complex (Pelmenschikov et al., 2002). Since none of the proposed intermediates have been trapped and spectroscopically or structurally characterized when using natural substrates, both mechanisms remain controversial. Based on spectroscopic and rapid kinetic studies, a hybrid mechanism involving both methyl-Ni and methyl radical has been proposed (Dey et al., 2010a,b; Li et al., 2010). In order to perform mechanistic studies, it is crucial to have a reliable protocol to generate the active state of MCR.

It is rather straightforward to purify native MCR from methanogenic cells; however, it has been extremely difficult to isolate and maintain MCR in its active state, due to the low midpoint redox potential of the Ni(II)/Ni(I) couple of bound F_430_ (between -700 and -600 mV compared to the normal hydrogen electrode, NHE; Holliger et al., 1993). Without special treatment of the cells and addition of reductants and HSCoM (or analogs) to the purification buffers, 99% of the MCR activity is lost upon cell lysis (Gunsalus and Wolfe, 1980; Brenner et al., 1993). In the late 1970s and early 1980s, several protein fractions (components A1, A2, A3a, A3b, and C) and compounds [component B and catalytic amounts of ATP, Mg^2+^, flavin adenine dinucleotide (FAD), and F_420_] were used to activate MCR from *Methanothermobacter thermautotrophicus* *in vitro* (Gunsalus and Wolfe, 1980; Nagle and Wolfe, 1983). However, only 1–5% of the MCR activity can be recovered by this activation.

In 1991, Thauer and colleagues found that the *M. marburgensis* MCR could be isolated in a much more active form (up to 50% of the *in vivo* activity) when cells are pre-incubated with 100% H_2_ and CH_3_-SCoM (or HSCoM) before lysis and purified under strict anaerobic conditions in the presence of Ti(III) citrate and HSCoM (Rospert et al., 1991a). CH_3_-SCoM and HSCoM were shown to stabilize both the activity and the EPR signal of MCR_red1_ (Rospert et al., 1991a). Since then, H_2_-dependent activation has been used to purify MCR_red1_ and 50–90% MCR_red1_ is routinely obtained (Li et al., 2010; Duin et al., 2011). Thus, although the steps involved in MCR activation are not known, it is clear that MCR activation involves a reductive activation to the Ni(I) state (Rospert et al., 1991b; Dey et al., 2006).

Here we report that carbon monoxide, which is a stronger reductant than H_2_, can activate MCR *in vivo* and in cell extracts of *M. marburgensis*. The rate of CO-dependent activation (130 min^-1^) is about 15-fold faster than H_2_-dependent activation (8 min^-1^). Furthermore, CO can activate MCR to a higher percentage. We observe no activation of MCR when cells are treated with formate, another strong reductant, using similar conditions as with CO or H_2_.

## MATERIALS AND METHODS

### BIOCHEMICALS, GAS MIXES, AND CELL GROWTH

All buffers, media ingredients, and other reagents were acquired from Sigma-Aldrich (St. Louis, MO, USA) and, unless otherwise stated, were of the highest purity available. Solutions were prepared using Nanopure deionized water. N_2_ (99.98%), CO (99.99%), argon (99.8%), H_2_/CO_2_ (80%/20%), and ultrahigh purity H_2_ (99.999%) were obtained from cryogenic gases (Grand Rapids, MI, USA). Ti(III) citrate solutions were prepared from a stock solution of 200 mM Ti(III) citrate, which was synthesized by adding sodium citrate to Ti(III) trichloride (30 wt% solution in 2 N hydrochloric acid; Acros Organics, Morris Plains, NJ, USA) under anaerobic conditions and adjusting the pH to 7.0 with sodium bicarbonate (Zehnder and Wuhrmann, 1976). The concentration of Ti(III) citrate was determined routinely by titrating a methyl viologen solution. *M. marburgensis* (formally *M. thermoautotrophicum* strain Marburg) was obtained from the Oregon Collection of Methanogens catalog as OCM82. *M. marburgensis* was cultured on H_2_/CO_2_ (80%/20%) at 65°C in a 14-L fermentor (New Brunswick Scientific Co. Inc., New Brunswick, NJ, USA). Culture media were prepared as previously described (Kunz et al., 2006) with a slight modification of the sulfur and reducing source, by adding 50 mM sodium sulfide (instead of H_2_S gas) at a flow rate of 1 ml min^-1^ during the entire growth period.

### ACTIVATION OF MCR IN WHOLE CELLS BY H_2_, CO, OR FORMATE

**FIGURE 1:**
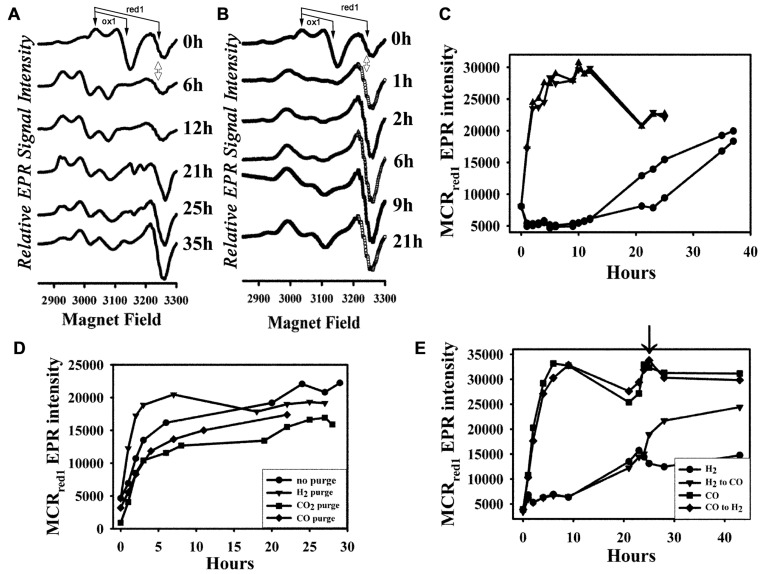
**Activation of MCR by H_2_ and CO *in vivo***. **(A)** EPR spectra of the H_2_-activated cell samples at different time points; **(B)** EPR spectra of the CO-activated cell samples at different time points; **(C)** plot of intensity of the MCR_red1_ EPR signal (the “S”-shaped derivative signal at ~3250 G, double-sided arrow) over time with duplicate H_2_- (circles) and CO-(triangles) activated cell samples; **(D)** plot of MCR_red1_ EPR intensity over time with CO activation with no initial purge (circles) in the fermentor, or initially purged with H_2_ (triangle), CO_2_ (square), or CO (diamond) in the fermentor; **(E)** plot of MCR_red1_ EPR intensity over time of treatment (as in **C**) with H_2_ (circles) and CO (triangles), arrow indicates the switch of gases. The features of the MCR_ox1_ and MCR_red1_ signals are depicted with arrows to demarcate their *g* values.

Solutions were prepared and all manipulations were performed under strictly anaerobic conditions in a Vacuum Atmospheres (Hawthorne, CA, USA) anaerobic chamber maintained under nitrogen gas at <1 ppm of oxygen (monitored continually by a Teledyne detector). Cells were grown under H_2_/CO_2_ as described (Dey et al., 2010a) and, before harvest, the cells were treated with H_2_ for 30 min in the fermenter (different gases were used in the experiments shown in **Figure 1D**). Then, the cells were harvested, transferred to the anaerobic chamber, and resuspended in 50 mM Tris, pH 7.6, 10 mM HSCoM, 0.1 mM Ti(III) citrate, and aliquoted into 150 ml serum-stoppered anaerobic bottles, as described (Dey et al., 2010a; Li et al., 2010). The headspaces of the vials containing the resuspended cells were then purged with CO or H_2_ for 10 min, or at timed intervals right before samples were taken for analysis. This second (and all subsequent) purge was also omitted for the formate-activation experiment as described in the next paragraph to ensure that the gas treatment did not interfere with the formate incubation. To facilitate equilibration of the gas with the solution, the vials were shaken every 2 min. In most experiments, the cell suspension was transferred into smaller vials, where the headspace was purged again for 10 min with the test gas. Purges with H_2_ were done inside of the chamber, while, for safety reasons, purges with CO were done in the fume hood outside of the chamber. To remove trace amounts of oxygen in the H_2_ or CO line for aerating the serum vials, the line was fitted with a high pressure Oxy-Trap oxygen scrub (Alltech, Deerfield, IL, USA) followed by an indicating Oxy-Trap (Alltech) to monitor the efficiency/remaining capacity of the oxygen scrub. Between the purges, the serum vials were inverted in a water bath in the anaerobic chamber to minimize any gas escape. For monitoring the conversion of MCR to the MCR_red1_ state, 200 μl samples were removed at different time points to be analyzed by EPR spectroscopy.

To determine whether formate activates MCR, the same protocol outlined above was followed (including the first 30 min H_2_ purge in the fermenter), except that sodium formate was added directly to the cell suspension to a final concentration of 100 or 500 mM (pH did not change in the Tris buffer that is described above for resuspending cells). Thus, there was only one gas purge in this experiment. To inhibit carbon monoxide dehydrogenase (CODH) activity, NaCN was added to the cell suspension to a final concentration of 0.2 or 0.4 mM before aerating with CO. Then, the cell suspension was incubated as described above.

### SPECTROSCOPY OF MCR

UV-visible spectra of MCR were recorded in the anaerobic chamber using a diode array spectrophotometer (model DT 1000A, Analytical Instrument Systems, Inc., Flemington, NJ, USA). All samples for EPR spectroscopy were prepared in 50 mM Tris–HCl buffer, pH 7.6, in a Vacuum Atmospheres anaerobic chamber maintained under nitrogen gas at <1 ppm of oxygen. EPR spectra were recorded on a Bruker EMX spectrometer (Bruker Biospin Corp., Billerica, MA, USA), equipped with an Oxford ITC4 temperature controller, a Hewlett-Packard model 5340 automatic frequency counter, and Bruker gaussmeter. Unless otherwise noted, the EPR spectroscopic parameters were: temperature, 70 K; microwave power, 10 mW; microwave frequency, 9.43 GHz; receiver gain, 2 × 10^4^; modulation amplitude, 10 G; and modulation frequency, 100 kHz. Double integrations of the EPR spectra were performed and referenced to a 1-mM copper perchlorate standard.

### PROTEIN PURIFICATION

The purification of MCR_red1_ from *M. marburgensis* was performed under strictly anaerobic conditions, as described previously (Kunz et al., 2006). The amount of MCR_red1_ was calculated according to the UV-visible and EPR spectra (Kunz et al., 2006; Li et al., 2010). Protein concentration was determined by Rose Bengal assay (Elliott and Brewer, 1978) with lysozyme as the standard.

### ENZYME ACTIVITY ASSAYS

Methyl-coenzyme M reductase assays for methane formation from methyl-CoM and CoBSH were performed at 60°C, basically as described by Kunz et al. (2006). The standard assay mixture contained 10 mM ^14^CH_3_-SCoM, 0.1 mM CoBSH, 1.8 mM aquacobalamin, 20 mM Ti(III) citrate, and 0.5 M Tris (pH 7.2) in a final volume of 0.2 ml. The reaction was started by adding MCR. MCR activity was measured by following the time-dependent loss of radioactivity from the methyl group of ^14^CH_3_-SCoM, which is converted to highly volatile and insoluble [^14^C] methane (Kunz et al., 2006). Rates of methane formation were calculated from the linear portion of the time course. One unit of MCR activity is equal to 1 μmol of methane produced per minute.

To determine formate dehydrogenase (FDH), hydrogenase, and CODH activities, *M. marburgensis* cell extracts were prepared under anaerobic conditions as described (Kunz et al., 2008). All assays were performed in a 1-ml sealed solution at 60°C. The anaerobic assay mixture contained 20 mM methyl viologen, 50 mM Tris, pH 7.5, and cell extract. For the hydrogenase and CODH assays, oxygen-free hydrogen gas or carbon monoxide (99.99%), respectively, was bubbled into the assay mix in a serum-stopped cuvette for 5 min (~1 mM of each gas in the solution) before enzyme was added to start the reaction. In the FDH assay, 20 mM formate was used and either 20 mM methyl viologen or 10 mM NADP^+^ was included as an electron acceptor. The activities were determined by following the reduction of methyl viologen at 578 nm with an extinction coefficient of 9.78 mM^-1^ cm^-1^ or the reduction of NADP^+^ at 340 nm with an extinction coefficient of 6.27 mM^-1^ cm^-1^.

## RESULTS

### GENERATION OF MCR_red_1 *IN VIVO* BY CO

To date, the most effective way to activate MCR has been to incubate the cell suspension under a H_2_ atmosphere (Rospert et al., 1991a; Kunz et al., 2006). While this is effective for generating MCR_red1_ from organisms like *M. marburgensis*, for some methanogens, like *Methanosarcina acetivorans*, H_2_ activation is inefficient because the hydrogenases are weakly expressed (Guss et al., 2009; Wang et al., 2011). Therefore, there must be other pathways for *in vivo* activation of MCR. We hypothesized that the low-potential reductant, CO, with a standard reduction potential (vs. NHE for the CO_2_/CO couple) of -520 mV (in comparison to -420 mV for that of 2 H^+^/H_2_; Lehn and Ziessel, 1982) should be able to activate MCR.

To measure the effectiveness of CO in activating MCR, we purged the *M. marburgensis* culture for 30 min with H_2_ in the fermentor, harvested the cells, resuspended the cells in buffer and incubated the cell suspensions at 32°C in a water bath under a CO or H_2_ atmosphere. The whole-cell EPR spectra of the H_2_- and CO-treated cells at different time points after initiating H_2_ or CO gas purge are compared in **Figures 1A,B**, respectively. The activity of MCR is linearly related to the MCR_red1_ EPR signal, with *g* values at 2.24 and 2.065; thus EPR spectroscopy is a convenient method to assess the level of MCR activation achieved. It also reveals various other EPR-active forms of MCR that are present during the treatment – for example, MCR_ox1_, which is an oxidized form of MCR that has a distinct EPR spectrum (*g* values at 2.23 and 2.16), can be converted into the MCR_red1_ state by the addition of low-potential reductants like Ti(III) citrate, and MCR_red2_, a Ni(I) state that is formed when the enzyme in the MCR_red1_ form is treated with HSCoM and CoBSH (Mahlert et al., 2002). Therefore, we feel that assessing the level of MCR_red1_ is the most accurate indicator of the level of activation achieved. The spectra of the cell suspension before the CO/H_2_ purge reveal about two to three times more MCR_ox1_ than MCR_red1_, very little MCR_red2_, and the remainder in the EPR-silent Ni(II) form. For the H_2_-purged cells, after ~1 h of incubation, MCR_ox1_ disappeared as MCR_red2_ increased; then, over the next ~20 h, MCR_red1_ appeared. Following a ~10 h lag time, the rate constant for H_2_-dependent activation was 8 min^-1^. The amount of MCR_red1_ formed at the end of activation, when compared with the amount of MCR_ox1_ in the beginning, varied slightly among enzyme preparations.

In contrast, when cells were incubated with CO during the timed purge, within 1 h, MCR underwent rapid activation to the MCR_red1_ state without a noticeable lag time or intermediacy of MCR_red2_. The enhanced activation by CO was noticeable by eye – the cell suspension began turning green immediately after aerating with CO. However, for H_2_-dependent activation, this color development required overnight incubation. At the end of activation (when the whole-cell EPR signal reached maximum intensity), the CO-activated cell suspension was also greener than that of the H_2_-activated cells (data not shown). Quantitative analysis of the activation confirmed the visual observations. The rate constant for CO-dependent activation was 130 min^-1^, which is over 15-fold faster than that observed for activation by H_2_; furthermore, the amount of accumulated MCR_red1_ was ~twofold higher than with H_2_ (**Figure 1C**). Furthermore, with CO activation, the amount of MCR_red1_ formed at the end of activation was approximately two times more than the initial amount of MCR_ox1_, indicating that CO activation converts the MCR_silent_ form to MCR_red1_.

As described above, the CO-dependent activation experiments shown in **Figures 1B,C** involved an initial 30 min H_2_ purge in the fermentor. To test whether the initial H_2_ treatment is required, we purged the cell suspension with pure H_2_, CO, or CO_2_ in the fermentor, or omitted the initial 30-min purge before harvesting, and then bubbled the cell suspension with CO after resuspending the cells in Tris–HCl buffer as above. The initial EPR spectra significantly differed with different purge conditions. MCR_ox1_ was only observed when the cells were initially purged with H_2_ in the fermentor, while MCR_red1_ was only noticeable with the CO treatment or when the initial H_2_ purge was omitted. On the other hand, MCR appears to be completely in a Ni(II)-silent form when cells were initially purged with CO_2_, because no detectable MCR signal was present. However, MCR underwent activation in all these conditions (i.e., H_2_, CO, CO_2_, no initial purge) after treating the cell suspensions with CO, and the activation rates and the final levels of activation were similar (**Figure 1D**). Thus, an initial H_2_ purge is not required for CO-dependent activation of MCR. This experiment also clearly shows that the EPR-silent Ni(II)-MCR forms undergo conversion to MCR_red1_ by CO *in vivo*.

We also tested the effect of addition of CO to the H_2_-purged cells. **Figure 1E** shows that treatment with CO can further activate MCR in H_2_-purged cells to nearly reach the level of MCR_red1_ observed in CO-activated cells. On the other hand, treatment with H_2_ does not increase the MCR_red1_ level in CO-activated cells. These experiments indicate that H_2_- and CO-dependent activations pathways converge after the initial transfer of electrons to the acceptor of reducing equivalents from hydrogenase or CODH.

### EFFECT OF TEMPERATURE ON CO-DEPENDENT MCR ACTIVATION

**FIGURE 2:**
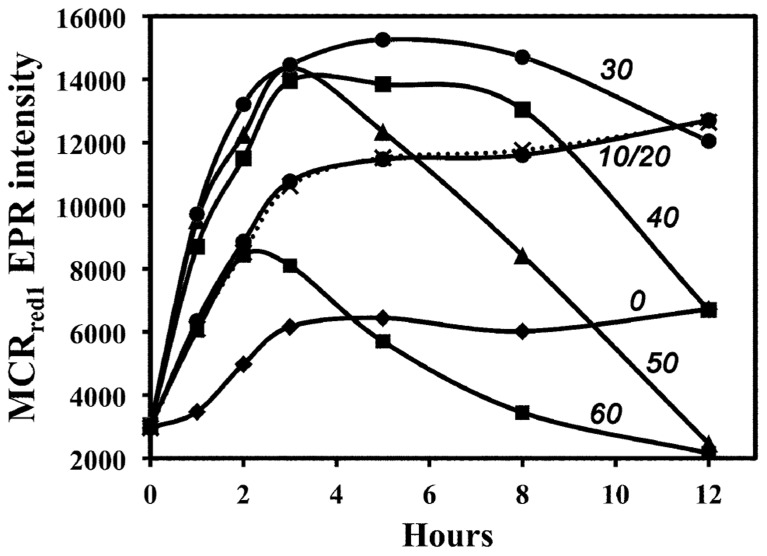
**Temperature dependence of *in vivo* MCR activation in whole cells by CO**. Activation temperatures (°C) are shown beside the relevant line.

H_2_-dependent activation of MCR is very sensitive to the incubation temperature; it is most effective at 30–33°C (Kunz et al., 2006). To test whether CO-dependent MCR activation is also temperature-dependent, we incubated the cell suspension with CO at different temperatures and followed the whole-cell EPR spectrum over 12 h. As shown in **Figure 2**, CO-dependent activation, like the H_2_-dependent process, is most effective at ~30°C. The complicating factor in these experiments is the inactivation of MCR, which occurs markedly faster as the temperature increases (Horng et al., 2001). At high temperatures (40–60°C), MCR_red1_ decays after 2 h of CO-dependent activation, while, at lower temperatures (10–20°C), activation by CO occurs more slowly, but, because MCR_red__1_ decays more slowly, this form of the enzyme is more stable and accumulates to higher levels.

### CODH ACTIVITY REQUIREMENT FOR CO-DEPENDENT ACTIVATION OF MCR

**FIGURE 3:**
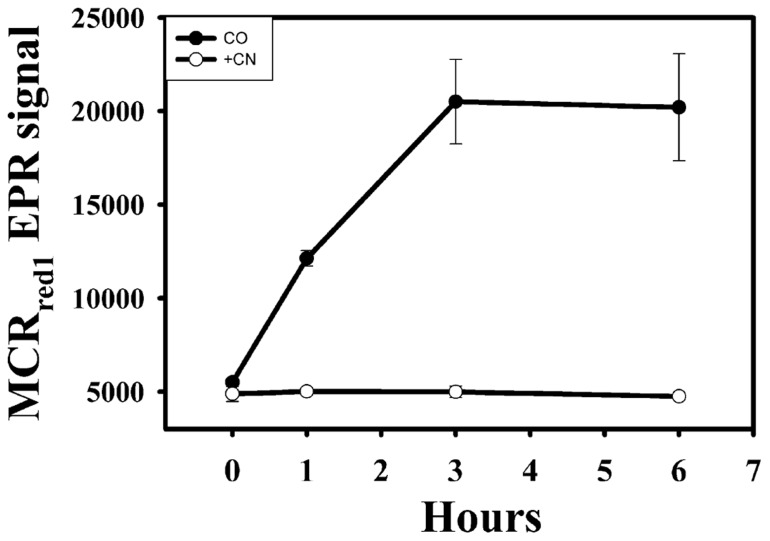
**Activation of MCR by CO in the presence of CODH inhibitor, CN^-^**. Filled circles, CO-purged samples; open circles, CO-purged samples in the presence of 0.2 mM CN^-^.

To test whether CODH activity is involved in CO-dependent activation of MCR, CN^-^, which is a competitive slow-binding inhibitor of CODH with a *K*_i_ for CODH inhibition in the low micromolar range (Ragsdale et al., 1983; Ha et al., 2007), was added to the cell suspension. As shown in **Figure 3**, 0.2 mM CN^-^ blocked the CO-dependent activation of MCR, indicating that CODH activity is required for CO-dependent activation of MCR. In contrast, 0.2–0.4 mM CN^-^ does not block H_2_-dependent activation of MCR (data not shown). We measured a specific activity of 3–5 U mg^-1^ of CODH in cell extracts, which is significantly lower than that of hydrogenase (~80 U mg^-1^), but is sufficient to drive the activation of MCR.

### PROPERTIES OF THE PURIFIED MCR FROM CO-ACTIVATED CELLS

**FIGURE 4:**
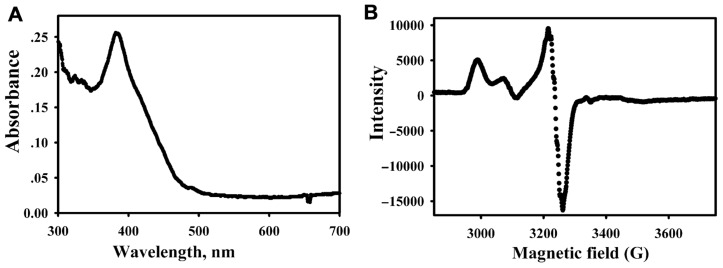
**Spectra of the purified MCR (9.4 μM heterotrimer, 1.3 mg ml^-1^) from CO-activated cells**. UV-visible **(A)** and EPR spectrum **(B)** of MCR purified from CO-activated cells.

Methyl-coenzyme M reductase was purified from cells that had been undergone a 5-h incubation with CO. Based on the UV-visible (**Figure 4A**) and EPR spectra (**Figure 4B**) of the purified enzyme, MCR was about ~80% active. Based on the enzymatic assay for the conversion of methyl-SCoM to methane, the specific activity of the CO-activated enzyme was calculated to be 89 ±10 U mg^-1^, indicating 89% conversion to the active MCR_red1_ state. Thus, the CO-dependent activation is highly efficient in producing a stable Ni(I) form of the enzyme.

### FORMATE CANNOT ACTIVATE MCR

**FIGURE 5:**
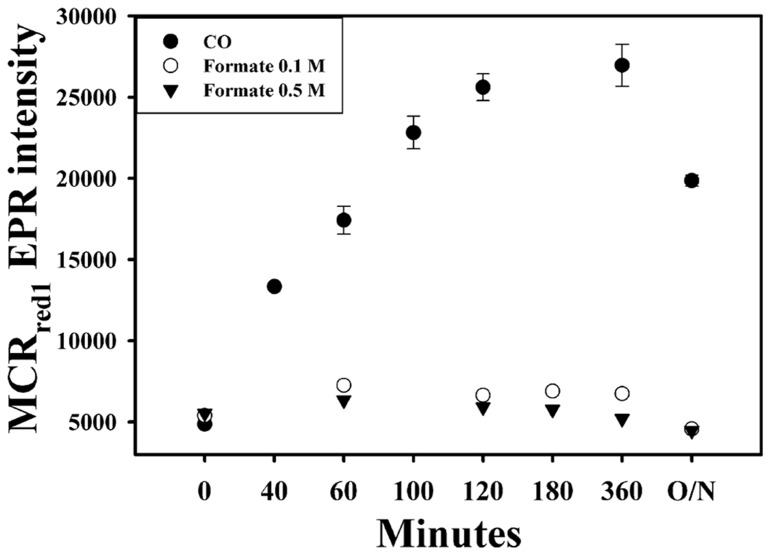
***In vivo* activation of MCR with CO (filled circles), 0.1 M formate (open circles), and 0.5 M formate (triangles).** O/N, overnight.

The standard redox potential of the CO_2_/HCOOH half-cell (-430 mV, Eq. 2) is equivalent to that of the 2H^+^/H_2_ half-cell (Eq. 3; Scherer and Thauer, 1978). Furthermore, the FDH gene (*fdh*) is present in the *M. marburgensis* genome and, based on sequence identity, is thought to use F_420_ as an electron donor (Liesegang et al., 2010; Kaster et al., 2011a). Thus, we expected that formate could also activate the *M. marburgensis* MCR. However, as shown in **Figure 5**, we did not observe any increase in the level of MCR_red1_ upon incubation of the cell suspension for over 20 h with formate. Correspondingly, we measured only a very low specific activity of FDH in cell extracts of *M. marburgensis* (3–4 mU mg^-1^, using NADP^+^ as electron acceptor and 2–3 mU mg^-1^, using MV), which is consistent with earlier reported values of 3–8 mU mg^-1^, depending on the electron acceptor and the pH of the assays (Tanner et al., 1989). We assayed using both NADP^+^ and MV, which is an electron acceptor for the F_420_-dependent enzyme, which the *M. marburgensis* enzyme is predicted to be (Kaster et al., 2011a). There are two forms of FDH: a tungsten- and a molybdenum-containing enzyme. The growth medium that we used has 1 μM molybdenum, but no tungsten (Kunz et al., 2006); however, addition of 1 μM tungsten and 1.1 μM selenium to the growth medium for *M. marburgensis*, also did not lead to activation of MCR in the presence of formate (data not shown). Apparently, the low level of FDH activity present in *M. marburgensis* is not sufficient to drive activation of MCR.

$$\begin{array}{cc} \begin{array}{c} {\text{CO}}_{2}+{2\text{H}}^{+}+{2\text{e}}^{-}\to \text{HCOOH }{\text{E}}^{\theta }=-0.43\text{V } \end{array} & \left(2\right) \end{array}$$

$$\begin{array}{cc} {2\text{H}}^{+}+{2\text{e}}^{-}\to {\text{H}}_{2}\,{\text{E}}^{\theta }=-0.42\text{V} & \left(3\right) \end{array}$$

$$\begin{array}{cc} {\text{CO}}_{2}+{2\text{H}}^{+}+{2\text{e}}^{-}\to \text{CO}+{\text{H}}_{2}\text{O }{\text{E}}^{\theta }=-0.52\text{V} & \left(4\right) \end{array}$$

## DISCUSSION

In order to study the mechanism of methane formation, it is crucial to develop an effective protocol for activation of MCR, the rate-limiting enzyme in this process. MCR has a number of different states, some of which have been detected *in vivo* (Albracht et al., 1988; Rospert et al., 1991a; Becker and Ragsdale, 1998) and others that have been observed *in vitro* (Mahlert et al., 2002; Finazzo et al., 2003; Duin et al., 2004; Kern et al., 2007; Harmer et al., 2008). Among these forms, only the Ni(I)-MCR_red_ (red1 and red2) forms are active and there is evidence supporting (Kunz et al., 2006; Dey et al., 2007, 2010a) and questioning (Ghosh et al., 2001; Pelmenschikov et al., 2002; Pelmenschikov and Siegbahn, 2003) the catalytic relevance of the methyl-Ni(III) state.

Without treating cells before they are harvested, MCR is isolated in the inactive Ni(II) state. This is the stable state of the enzyme and, except for one structure of the methyl-Ni(III) state (Cedervall et al., 2011), it is the only form whose crystal structure is known. There exist no published methods for conversion of any of the Ni(II)-MCR_silent_ forms into MCR_red1_ *in vitro*. Thauer and colleagues discovered that MCR_ox1_, an inactive EPR-active state that has been termed the “ready” state, can be generated by purging the cells with 80% N_2_/20% CO_2_ before harvesting; then MCR_ox1_ can be isolated and reduced to active MCR_red1_ *in vitro* by addition of the reductant, Ti(III) citrate (Goubeaud et al., 1997). In lieu of the gas purging protocol, formation of MCR_ox1_ can be optimized by adding sulfide to the culture prior to harvesting (Becker and Ragsdale, 1998). Then, as with the gas-switching method, the relatively stable MCR_ox1_ state of the enzyme can be purified and quantitatively converted to MCR_red1_ by treating with Ti(III) citrate.

Here we show that CO, like H_2_, can activate the *M. marburgensis* MCR *in vivo*. When cells are purged with CO (**Figure 1C**), the whole-cell EPR signal of MCR_red1_ increases much more rapidly than that of H_2_-purged cells. The more rapidly MCR can be activated the better, because oxidants are present in the whole cells and in extracts that oxidize the Ni(I) state back to inactive Ni(II). However, once the enzyme is purified and maintained under anaerobic conditions, the MCR_red1_ state is rather stable. This CO-purging protocol yields purified MCR that is 80–90% MCR_red1_, as measured by the UV-visible and EPR spectral intensities and the specific activity.

Because growth of *M. marburgensis* on CO has not been reported, we were somewhat surprised that CO can activate MCR. However, there is a copy of the CODH gene (MTBMA c02870-02930) in the *M. marburgensis* genome (Liesegang et al., 2010; Kaster et al., 2011a) and relatively high CODH activities are measured in the cell extracts. This enzyme would be required for the autotrophic fixation of CO_2_ by *M. marburgensis* (Ferry, 2010). It is likely that it is this CODH activity that is required for CO-dependent activation of MCR, because we are unaware of other enzymes that can use CO as an electron donor and because CN^-^, a potent CODH inhibitor, blocks CO-dependent activation of MCR (**Figure 3**).

On the other hand, incubation of cells with formate under the same condition used for H_2_- and CO-dependent activation does not activate MCR. Although *M. marburgensis* can assimilate formate and has FDH activity, it is unlike the related strains, *Methanothermobacter thermautotrophicus* strain Z-245 and *Methanothermobacter wolfeii* (Nolling et al., 1993; Wasserfallen et al., 2000), in that it cannot grow on formate as an energy source (Kaster et al., 2011a). Mutants of *M. marburgensis* lacking FDH require formate for growth on H_2_ and CO_2_ (Tanner et al., 1989). Our experiments confirm earlier reports (Tanner et al., 1989) that the specific activity of FDH is very low – approximately 1000-fold lower than that of CODH, probably insufficient to support activation of MCR.

Thus, there are now two known electron sources that can reduce MCR to active MCR_red1_: H_2_ and CO. One possible activation mechanism involves H_2_ as an intermediate because CO-dependent H_2_ production has been reported in methanogens (Daniels et al., 1977; O’Brien et al., 1984), however, it is clear that this is not the pathway for CO-dependent activation of MCR because the rate of activation by CO is about 15 times faster than that with H_2_. Furthermore, the lag phase observed in H_2_-dependent activation is not present in the CO-dependent process. One might also argue that CO is an intermediate in the H_2_-dependent activation process and that the lag phase observed in H_2_-dependent activation is due to the time needed for formation of CO from H_2_ and CO_2_ in the suspensions. However, this possibility is ruled out by the fact that CN^-^ blocks CO-, but not H_2_-dependent activation of MCR. Yet, that CO further activates the H_2_-activated enzyme to reach the level of MCR_red1_ observed in CO-activated cells (but not vice versa – H_2_ hardly activates MCR in CO-activated cells) indicates that H_2_- and CO-dependent activations pathways converge after the initial electron transfer from hydrogenase or CODH. The reason for the faster and higher activation level of MCR by CO may be related to the standard redox potential of the CO_2_/CO half-cell (-520 mV, Eq. 4), which is approximately 100 mV more negative than that of the 2H^+^/H_2_ half-cell (Lehn and Ziessel, 1982; Drake et al., 2006; Thauer et al., 2008; Kaster et al., 2011b). According to Marcus theory, for a one-electron redox reaction, a 100-mV greater driving force would be expected to increase the rate of electron transfer by ~30-fold at 30°C. Accordingly, we observe that CO-dependent activation is about 15 times faster that the H_2_-dependent process. This driving force argument is also supported by our results that CO activation is faster than H_2_ even though hydrogenase activity in *M. marburgensis* cells is 20-fold greater than that of CODH.

Activation of MCR involves reduction of the nickel center of F_430_ to the Ni(I) state (Rospert et al., 1991b; Dey et al., 2006). The electron carrier(s) needed for activation must interface with CODH (or hydrogenase, when cells are activated with H_2_) and with MCR. Although the methanogenic CODH is recognized to interface with ferredoxin (Terlesky and Ferry, 1988), our results indicate that there are additional redox steps involved in activation that couple CO-reduced ferredoxin to MCR. These components are probably also required in the H_2_-dependent activation. However, the activation by H_2_ is likely to be more complex than that for CO because the midpoint potential for the Ni(II)/(I) couple of bound F_430_ (less than -600 mV) is significantly lower those of ferredoxin (approximately -450 mV) and 2 H^+^/H_2_ (-414 mV), and only slight lower than that of CO_2_/CO (-520 mV). Thus, some type of bioenergetic coupling is required for activation of MCR (at least in the case of H_2_-dependent activation). We speculate that this mechanism of activation might involve electron bifurcation, which has been seen to drive various uphill enzymatic reactions (Lie et al., 2012; Thauer, 2012).

Cell extracts of *M. thermoautotrophicus* have been shown to slowly catalyze the reduction of CO_2_ to methane only upon addition of CH_3_-SCoMor CoBS-SCoM (Gunsalus and Wolfe, 1977; Rouviére and Wolfe, 1988). This phenomenon is referred to as the RPG effect, which couples the first step in methanogenesis from CO_2_ and H_2_ to the reduction of CoBS-SCoM to the free thiolate cofactors. Kaster et al. (2011a) demonstrated that the MvhADG/HdrABC complex from hydrogenotrophic methanogens couples the endergonic reduction of ferredoxin (midpoint potential of -450 to -500 mV) with H_2_ to the exergonic reduction of CoBS-SCoM with H_2_. Furthermore, reduction of ferredoxin by H_2_ (-414 mV at 100% gas phase under 1 bar pressure) at pH7 occurs only if CoBS-SCoM is present – thus, this is a coupled and energy driven reaction. We speculate that electron bifurcation involving CoBS-SCoM may be involved in the uphill reductive activation of MCR. The need for coupling of reduction of ferredoxin to the reduction of CoBS-SCoM by H_2_ may explain why there is a lag phase in the H_2_-dependent activation of MCR.

## Conflict of Interest Statement

The authors declare that the research was conducted in the absence of any commercial or financial relationships that could be construed as a potential conflict of interest.
